# Serum concentrations of interleukin 18 and 25-hydroxyvitamin D_3_ correlate with depression severity in men with psoriasis

**DOI:** 10.1371/journal.pone.0201589

**Published:** 2018-08-09

**Authors:** Daniel Pietrzak, Aldona Pietrzak, Ewelina Grywalska, Paweł Kiciński, Jacek Roliński, Helena Donica, Kinga Franciszkiewicz-Pietrzak, Andrzej Borzęcki, Mateusz Socha, Jarosław Niedziałek, Dorota Krasowska

**Affiliations:** 1 1st Department of Anaesthesiology and Intensive Care, Medical University of Lublin, Lublin, Poland; 2 Department of Dermatology, Venereology and Pediatric Dermatology, Medical University of Lublin, Lublin, Poland; 3 Department of Clinical Immunology and Immunotherapy, Medical University of Lublin, Lublin, Poland; 4 Department of Experimental Hematooncology, Medical University of Lublin, Lublin, Poland; 5 Department of Hematology, St. John's Cancer Center, Lublin, Poland; 6 Department of Biochemical Diagnostics, Medical University of Lublin, Lublin, Poland; 7 Department of Surgical Oncology, Medical University of Lublin, Lublin, Poland; 8 Department of Hygiene, Medical University of Lublin, Lublin, Poland; 9 Department of Internal Medicine and Cardiology 1st Military Hospital in Lublin; Lublin, Poland; 10 Jarosław Niedziałek—Individual Medical Practice, Lublin, Poland; Department of Psychiatry and Neuropsychology, Maastricht University Medical Center, NETHERLANDS

## Abstract

**Objective:**

Psoriasis and depression may have common mechanisms, such as systemic inflammation, dysfunction of the hypothalamic-pituitary-adrenal axis, and vitamin D_3_ deficiency. Among men with psoriasis, this study examined whether depression severity was associated with serum concentrations of different metabolic and inflammatory markers.

**Methods:**

The study included 85 men with psoriasis (mean age ± standard deviation [SD], 47 ± 14 years) and 65 men without psoriasis (mean age ± SD, 44 ± 13 years). In both groups, we measured the body mass index; blood pressure; and serum concentrations of lipids, uric acid, lipase, interleukins 6 and 18, cortisol, and 25-hydroxyvitamin D_3_. All participants completed the Beck Depression Inventory. Other variables analyzed included psoriasis duration, the Psoriasis Area Severity Index, and the percentage of body surface area affected by psoriatic lesions.

**Results:**

Compared with controls, patients with psoriasis had significantly greater depression severity, higher body mass indices, and higher serum concentrations of total cholesterol and interleukins 6 and 18; moreover, they had significantly lower serum 25-hydroxyvitamin D_3_ concentrations. In patients with psoriasis, depression severity correlated positively with psoriasis duration, the Psoriasis Area Severity Index, the percentage of body surface area affected by psoriatic lesions, and interleukin-18 concentration. In patients with psoriasis, depression severity correlated negatively with 25-hydroxyvitamin D_3_ concentration, but it did not correlate significantly with the serum concentrations of interleukin 6 and cortisol.

**Conclusions:**

High concentrations of interleukin 18 and low concentrations of 25-hydroxyvitamin D_3_ may be associated with depression severity in men with psoriasis. Thus, further studies should examine whether effective anti-inflammatory treatments or vitamin D_3_ supplementation can improve depression outcomes in these patients.

## Introduction

Psoriasis is diagnosed more and more often; its pathogenesis is complex and involves immune dysregulation and chronic systemic inflammation [1]. Due to this complex pathogenesis, psoriasis rarely presents in isolation, but rather co-occurs with other conditions, such as psychiatric diseases, metabolic syndrome, and alcohol and tobacco dependence [1–4]. Of all conditions that co-occur with psoriasis, depression is the most common, affecting up to 80% of patients with psoriasis [5–6]. In psoriasis, depression worsens both treatment outcomes and prognosis [7]; for example, it increases the risk of psoriatic arthritis [8]. We and other investigators showed that longer psoriasis duration and greater skin lesion severity are associated with depressed mood [9–14]. However, some studies did not support these observations [15–16]. A growing body of evidence suggests that psoriasis and depression may have common mechanisms, such as systemic inflammation [17–18], dysfunction of the hypothalamic-pituitary-adrenal (HPA) axis [19–21], and vitamin D_3_ deficiency [22–24]. It is hypothesised that such non-psychological factors may increase the risk of depression in patients with psoriasis, but the evidence supporting this hypothesis is lacking.

Thus, among men with psoriasis, we analyzed the relationship between depression severity and serum concentrations of interleukins (IL) 6 and 18 (both implicated in the pathogenesis of psoriasis), cortisol, and 25-hydroxyvitamin D_3_ [25(OH)D_3_].

## Patients and methods

### Participants

The study included 85 men with psoriasis (mean age ± standard deviation [SD], 47 ± 14 years) and 65 men without psoriasis, who served as controls (mean age ± SD, 44 ± 13 years). Because women with psoriasis have depression more often than do men with psoriasis, [25], we did not include women, to avoid potential confounding effects of sex. All participants were recruited between 2014 and 2016 at the Department of Dermatology, Venereology, and Pediatric Dermatology, Medical University of Lublin, Poland. The mean psoriasis duration was 18 ± 13 (SD) years, and the mean Psoriasis Area Severity Index (PASI) score was 17 ± 9 (SD) points. The controls received treatment due to diseases other than psoriasis, such as pigment nevi, mild acne, small filiform warts, and single fungal nail infections. The exclusion criteria were as follows: history of recent myocardial disease, renal insufficiency, severe systemic diseases with fever, psychiatric disorders, and previous or current treatment with biologicals or immunosuppressive agents. Neither the patients nor the controls received any medications that might have changed their mood. None of the patients received ultraviolet light therapy or vitamin D_3_ supplementation.

### Ethics

The study protocol was approved by the Local Bioethics Committee of the Medical University of Lublin (decision no. KE-0254/283/2014 of 30 October 2014), and written informed consent was obtained from all individual participants included in the study.

### Variables analyzed

During a routine visit to the clinic, each participant was asked to complete the Beck Depression Inventory (BDI). The BDI comprises 21 items, each scored on a four -point scale (from 0 to 3 points). The final score ranges from 0 to 63 points and is interpreted as no depression (0–11 points), mild depression (12–26 points), moderate depression (27–49 points), or severe depression (50–63 points). The BDI can be used to examine symptoms of depression over any period; typically, over the past month, which was also the case in our study. The BDI’s internal consistency is very good (Cronbach’s alpha, 0.91).

The explanatory variables were as follows: disease duration; PASI score; percentage of body surface area affected by psoriatic lesions (BSA); body mass index (BMI); systolic and diastolic blood pressure; and serum concentrations of total cholesterol, low-density lipoprotein (LDL) cholesterol, high-density lipoprotein (HDL) cholesterol, triglycerides (TG), uric acid, lipase, IL-6, IL-18, cortisol, and 25(OH)D_3_.

Whole-blood samples were collected in the morning, after an overnight fast. After 20 minutes of centrifugation at 1,000 × g, supernatant was separated from the blood samples and used for testing or stored at -80°C. The total cholesterol concentration was measured with a colorimetric method with cholesterol esterase and oxidase; HDL-cholesterol concentration, with a direct enzymatic-colorimetric method with polyethylene glycol (PEG)-modified cholesterol esterase and oxidase; and TG, with an enzymatic-colorimetric method with phosphoglycerol oxidase. The serum LDL-cholesterol concentration was calculated according to the Friedewald equation. The uric acid concentration was measured with an enzymatic-colorimetric method with uricase. The lipase concentration was measured with a colorimetric method with 1,2-O-dilauryl-rac-glycero-3-glutaric acid-(6-methylresorufin) ester as a chromogenic substrate. The Cobas Integra 400 analyzer (Roche Diagnostics, Tokyo, Japan) and commercially available reagents (Roche, Tokyo, Japan) were used for all these measurements.

The serum concentration of 25(OH)D_3_ was measured with an electro-chemiluminescence assay and the Cobas e601 analyzer (Roche Diagnostics, Tokyo, Japan). Enzyme-linked immunosorbent assays were used to measure the serum concentrations of cortisol (Cortisol ELISA, IBL International, Hamburg, Germany), IL-6 (Human IL-6 High Sensitivity ELISA Kit, Diaclone, Besancon, France), and IL-18 (Human IL-18 ELISA Kit, MBL, Nagoya, Japan). All ELISAs were read with a spectrophotometric plate reader (Power Wave XS, Bio-Tek, Winooski, United States).

### Statistical analysis

Normal distribution of continuous variables was checked with the Shapiro-Wilk test, and normally distributed variables were presented as means and SDs. The Student t-test for independent variables was used to compare continuous variables between two groups. The Pearson’s coefficient (r) was used to examine correlations between pairs of variables. The predictors of depression in patients with psoriasis were identified with an artificial neural network with MLP 7-9-1 structure and BFGS learning algorithm (SOS error function, activation function of the neurons in the hidden layer–tangent function and activation function of the neurons in the output layer–logistic function). We used analysis of variance (ANOVA) with the Tukey’s test for *post hoc* comparisons or the Kruskall-Wallis test to compare patients without depression, patients with mild depression, and patients with moderate depression. The Statistica 10 package (StatSoft, Tulsa, OK, United States) was used for all calculations; p < 0.05 was considered statistically significant.

## Results

Physical, clinical, and laboratory characteristics of the patients and controls are presented in Table 1. Compared with the controls, patients with psoriasis had significantly higher BDI scores, BMIs, and serum concentrations of total cholesterol, IL-6, and IL-18; they had significantly lower 25(OH)D_3_ concentrations. The concentrations of LDL-cholesterol, HDL-cholesterol, TG, uric acid, lipase, and cortisol did not differ between the groups.

**Table 1:** Characteristics of men with psoriasis and controls.

Parameter	Patients with psoriasis (n = 85) [mean ± SD]	Controls (n = 65) [mean ± SD]	p
Age (years)	47 ± 14	44 ± 13	NS
PASI	17 ± 9	-	-
Disease duration (years)	18 ± 13	-	-
BSA (%)	26 ± 19	-	-
BMI (kg/m^2^)	28 ± 5	26 ± 3	0.003
Systolic blood pressure (mm Hg)	1.3 x 10^2^ ± 0.1 x 10^2^	1.3 x 10^2^ ± 0.1 x 10^2^	NS
Diastolic blood pressure (mm Hg)	81 ± 10	80 ± 8	NS
Total cholesterol (mg/dl)	2.1 x 10^2^ ± 0.4 x 10^2^	1.9 x 10^2^ ± 0.4 x 10^2^	0.01
LDL-cholesterol (mg/dl)	1.2 x 10^2^ ±0.3 x 10^2^	1.3 x 10^2^ ± 0.4 x 10^2^	NS
HDL-cholesterol (mg/dl)	50 ± 13	54 ± 15	NS
TG (mg/dl)	1.6 x 10^2^ ± 0.8 x 10^2^	1.3 x 10^2^ ± 0.7 x 10^2^	NS
Uric acid (mg/dl)	6.1 ± 1.6	5.7 ± 1.1	NS
Lipase (U/l)	35 ± 19	32± 16	NS
Interleukin 6 (pg/ml)	4.3 ± 4.6	0.700 ± 0.005	<0.0001
Interleukin 18 (pg/ml)	2.8 x 10^2^ ± 1.2 x 10^2^	2.2 x 10^2^ ± 1 x 10^2^	0.006
Cortisol (ng/ml)	1.3 x 10^2^ ± 0.4 x 10^2^	1.3 x 10^2^ ± 0.4 x 10^2^	NS
25-hydroxyvitamin D_3_ (ng/ml)	12 ± 5	22± 9	<0.0001
Beck Depression Inventory (points)	13 ± 11	4.5 ± 4.4	<0.0001

BMI, body mass index; BSA, body surface area affected by psoriatic lesions; NS, non-significant (p>0.05); PASI, Psoriasis Area Severity Index; LDL, low-density lipoprotein; HDL, high-density lipoprotein; TG, triglycerides; SD, standard deviation

Among patients with psoriasis, the BDI scores correlated positively with the serum IL-18 concentration (r = 0.27, p = 0.0100), PASI scores (r = 0.46, p < 0.0001), BSAs (r = 0.46, p < 0.0001), and psoriasis duration (r = 0.43, p < 0.0001); the BDI scores correlated negatively with serum 25(OH)D_3_ concentrations (r = -0.23, p = 0.0200; Table 2).

**Table 2:** Pearson’s correlation coefficients (r) between Beck Depression Inventory scores and clinical characteristics of patients with psoriasis (n = 85).

Variables		r	p
Beck Depression Inventory scores	25-hydroxyvitamin D_3_ (ng/ml)	-0.23	0.02
	Interleukin 6 (pg/ml)	0.18	NS
	Interleukin 18 (pg/ml)	0.27	0.01
	Cortisol (ng/ml)	0.03	NS
	PASI	0.46	<0.0001
	BSA (%)	0.46	<0.0001
	BMI (kg/m^2^)	0.11	NS
	Disease duration (years)	0.43	<0.0001

BMI, body mass index; BSA, body surface area affected with psoriatic lesions; NS, non-significant (p>0.05); PASI, Psoriasis Area Severity Index

These findings were confirmed by the artificial neural network analysis, in which numerical weights for all variables that correlated significantly with BDI scores in patients with psoriasis were markedly higher than 1 (Table 3).

**Table 3:** Clinical and laboratory predictors of depression in patients with psoriasis–numerical weights of an artificial neural network.

Group	Factor	Numerical weight
Clinical predictors	Disease duration (years)	1.5
	PASI	4.8
	BSA (%)	1.2
Laboratory predictors	Interleukin 6 (pg/ml)	0.9
	Interleukin 18 (pg/ml)	2.8
	25-hydroxyvitamin D_3_ (ng/ml)	1.1
	Cortisol (ng/ml)	1.0

BSA, body surface area affected with psoriatic lesions; PASI, Psoriasis Area Severity Index

**Fig 1:**
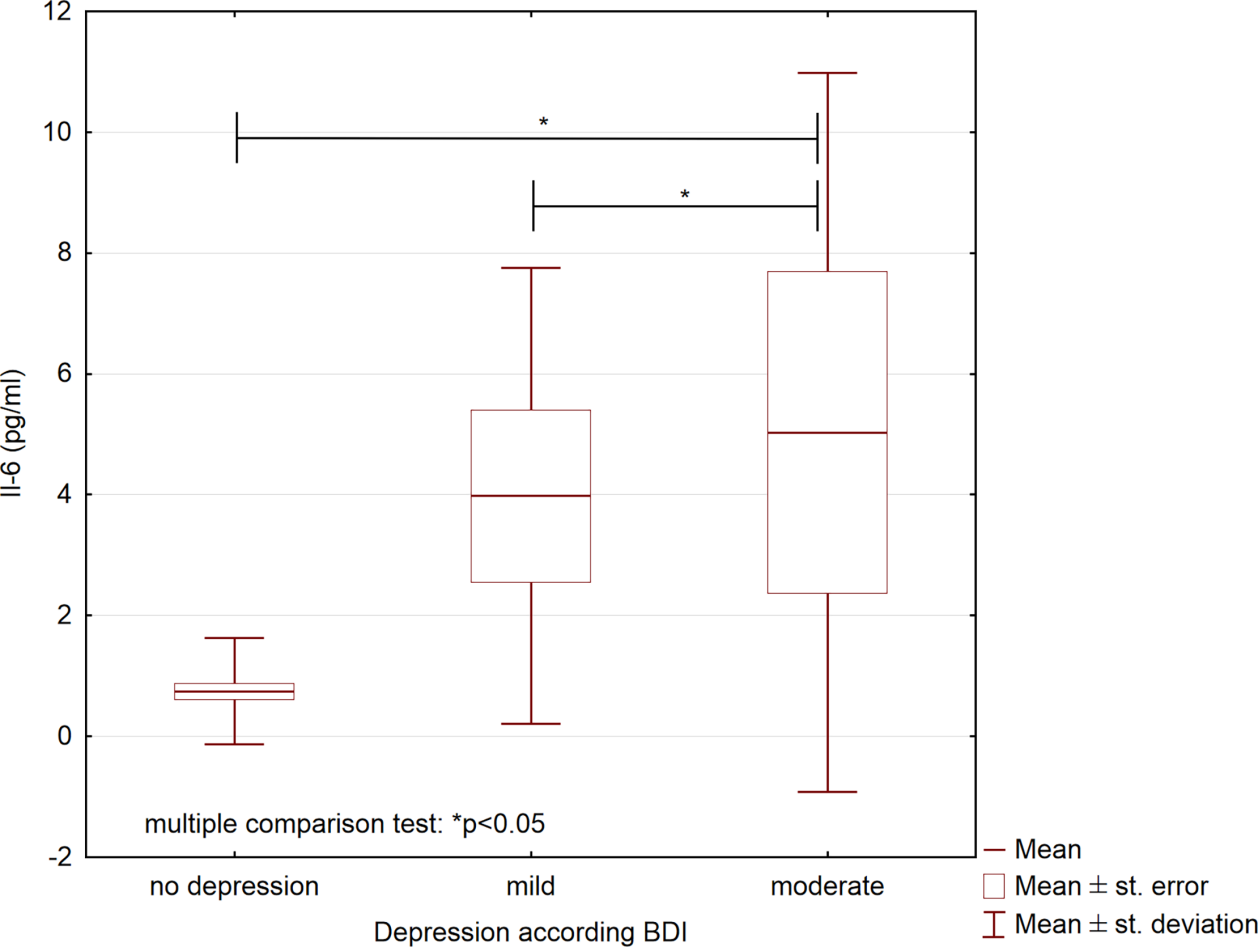
Serum concentrations of interleukin 6 in participants with different depression severity (N = 150).

**Fig 2:**
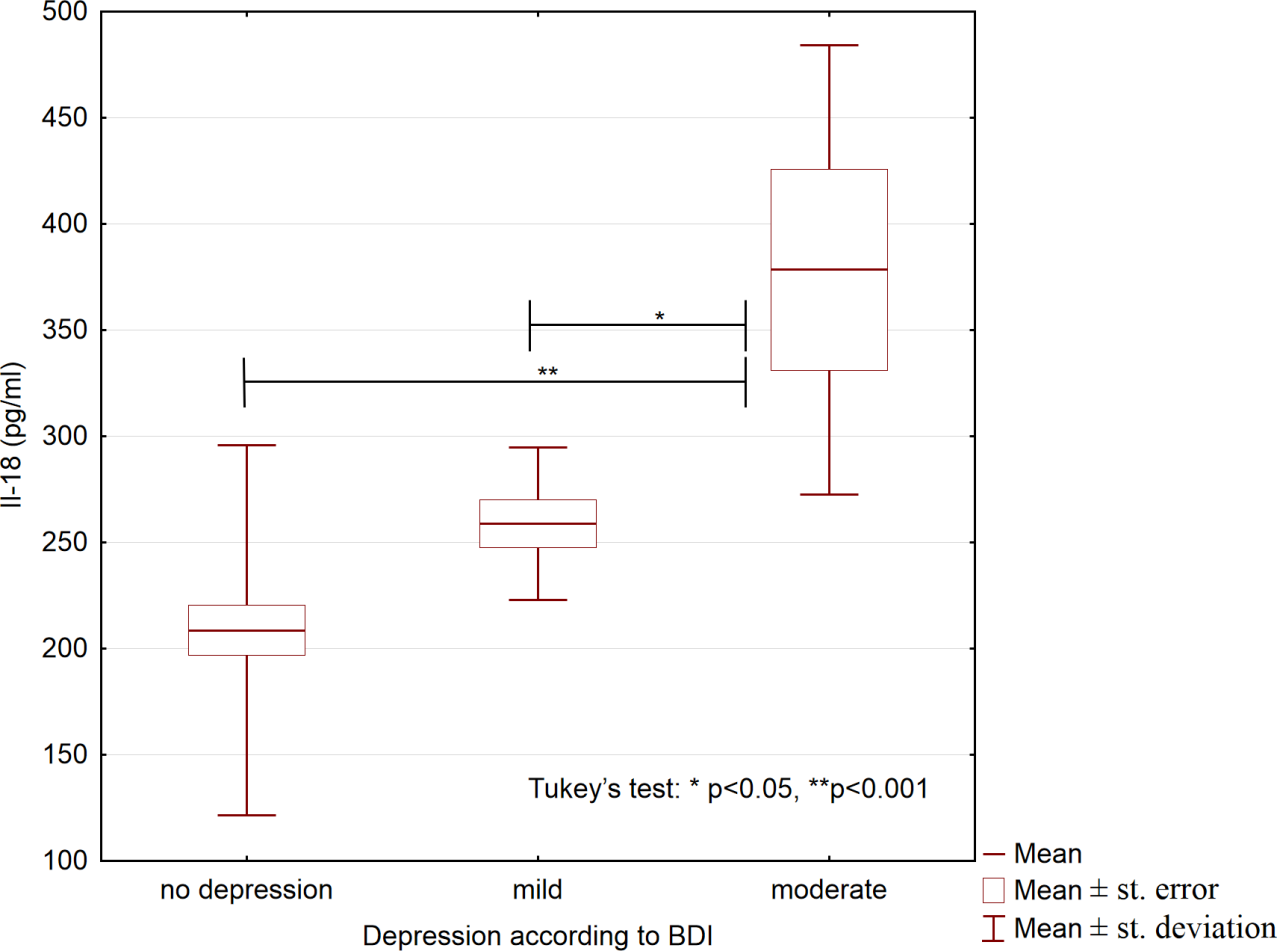
Serum concentrations of interleukin 18 in participants with different depression severity (N = 150).

**Fig 3:**
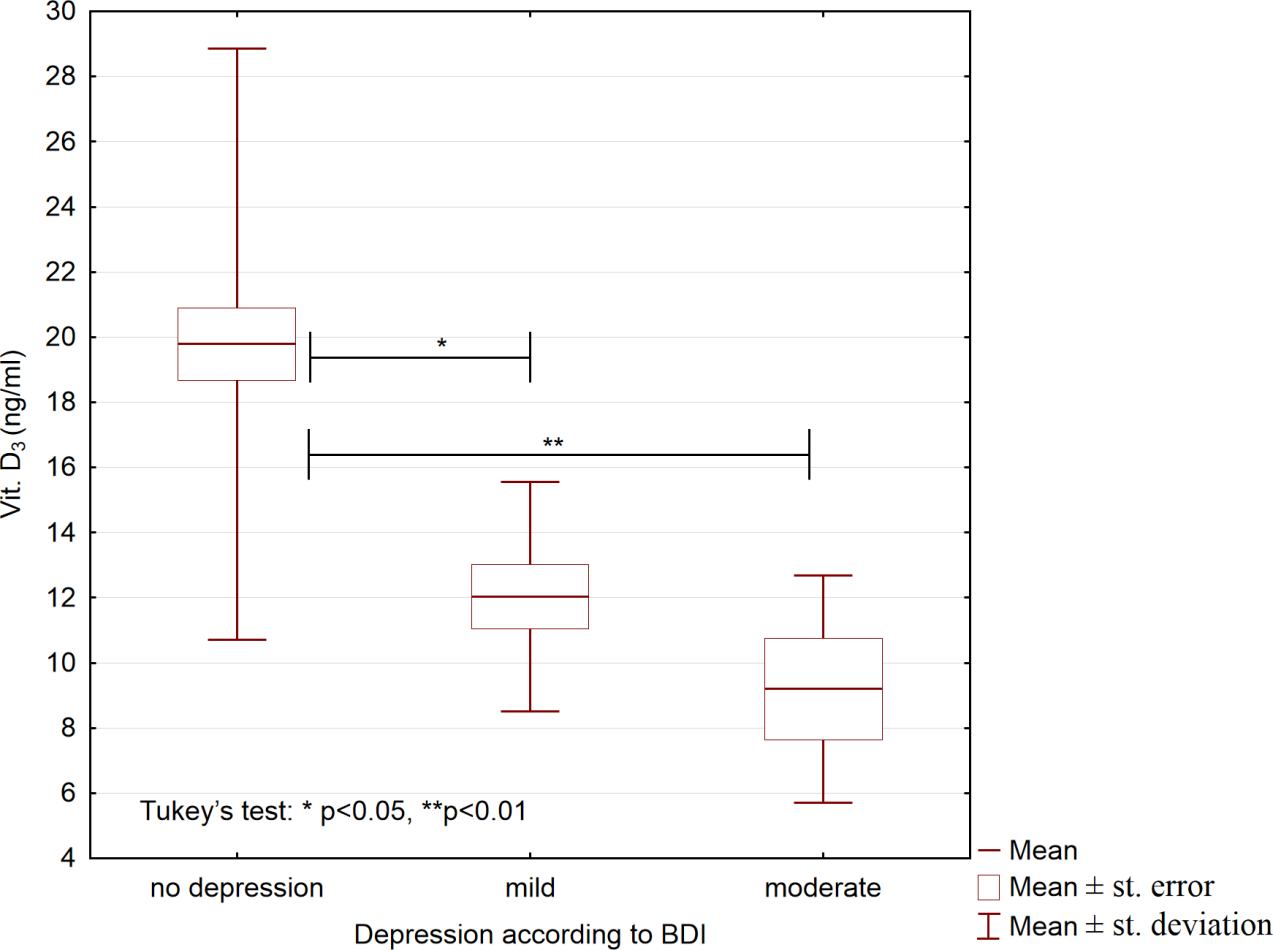
Serum concentrations of 25-hydroxyvitamin D_3_ in participants with different depression severity (N = 150).

We also studied whether the analyzed variables differed between participants who differed in depression severity. Based on the BDI scores, we allocated all participants, both patients and controls, to the three following groups: no depression (BDI score, 0–11; n = 112), mild depression (BDI score, 12–26; n = 25), and moderate depression (BDI score, 27–49; n = 13); no participants had severe depression (BDI score, 50–63 points). These groups differed significantly with respect to the concentrations of IL-6, IL-18, and 25(OH)D_3_ (Table 4). Compared to participants with mild depression and participants without depression, participants with moderate depression had higher serum concentrations of IL-6 and IL-18 (Figs 1 and 2), and lower 25(OH)D_3_ concentrations (Fig 3).

**Table 4:** Serum concentrations of interleukins 6 and 18, 25-hydroxyvitamin D_3_, and cortisol in all participants (N = 150) according to depression severity.

	No depression (mean ± SD; n = 112)	Mild depression (mean ± SD; n = 25)	Moderate depression (mean ± SD; n = 13)	p
**Interleukin 6** **(pg/ml)**	0.74 ± 0.88	3.9 ± 3.8	5.0 ± 6.0	0.001
**Interleukin 18 (pg/ml)**	2.1 x10^2^ ± 0.9 x 10^2^	2.6 x 10^2^ ± 0.4 x 10^2^	3.8 x 10^2^ ± 1.1 x 10^2^	<0.001
**25-hydroxyvitamin D**_**3**_ **(ng/ml)**	19.7 ± 8.9	12.0 ± 3.5	9.2 ± 3.5	<0.001
**Cortisol (ng/ml)**	1.2 x 10^2^ ± 0.4 x 10^2^	1.4 x 10^2^ ± 0.5 x 10^2^	1.2 x 10^2^ ± 0.1 x 10^2^	0.31

SD, standard deviation

**Fig 4:**
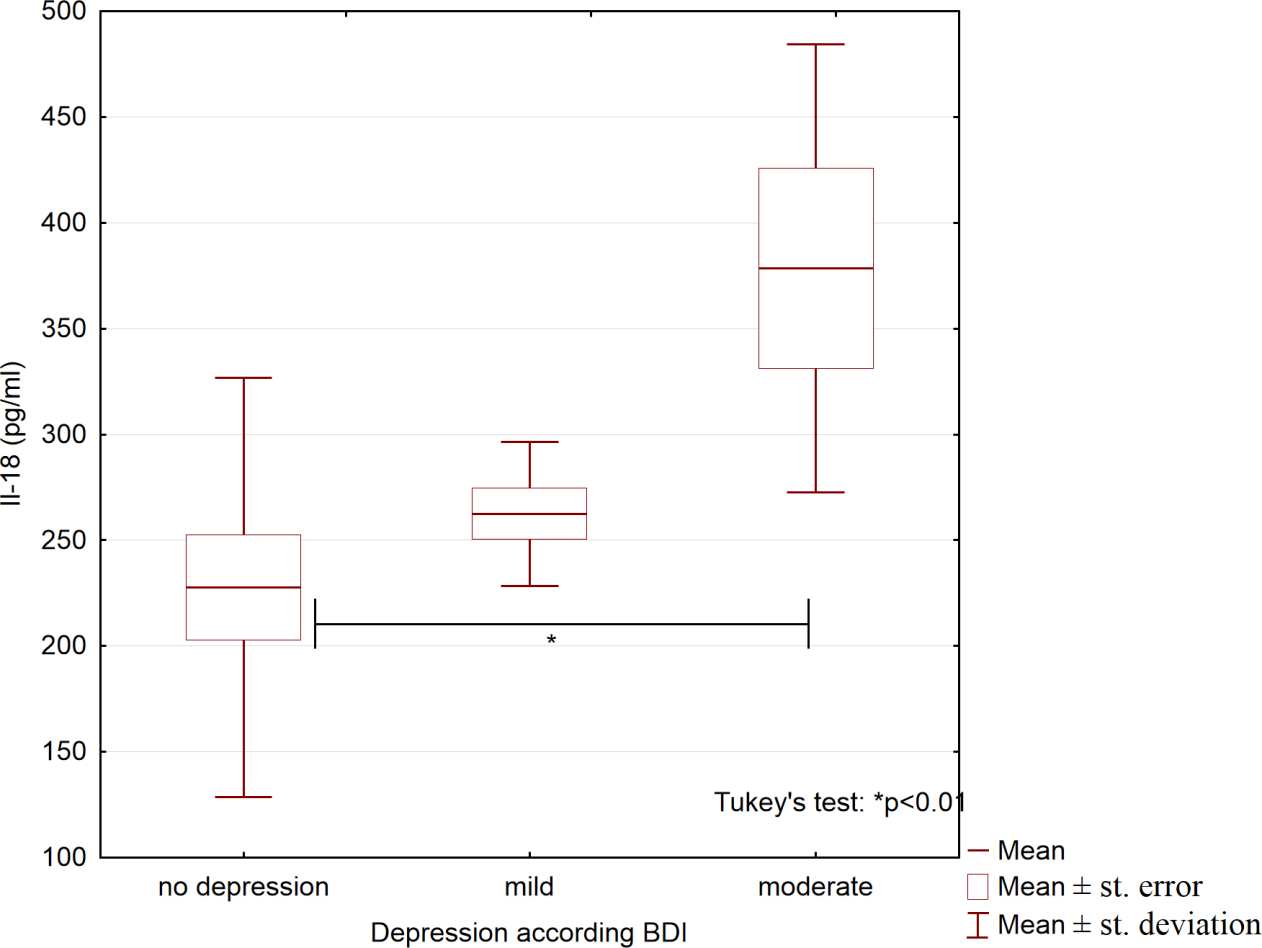
Serum concentrations of interleukin 18 in patients with psoriasis and different depression severity.

Moreover, we examined whether the variables analyzed differed between patients with psoriasis who had no depression (n = 52), mild depression (n = 20), and moderate depression (n = 13). Only the IL-18 concentration differed significantly between these groups (Table 5); compared with patients without depression, patients with moderate depression had significantly higher IL-18 concentrations (Fig 4).

**Table 5:** Serum concentrations of IL-6, IL-18, 25-hydroxyvitamin D_3_, and cortisol in patients with psoriasis (n = 85) according to depression severity.

	No depression (mean ± SD; n = 52)	Mild depression (mean ± SD; n = 20)	Moderate depression (mean ± SD; n = 13)	p
**Interleukin 6 (pg/ml)**	1.3 ± 0.8	3.9 ± 4.1	5.0 ± 6.0	0.25
**Interleukin 18 (pg/ml)**	2.3 x 10^2^ ±1.0 x 10^2^	2.7 x 10^2^ ± 0.3 x 10^2^	3.8 x 10^2^ ± 1.1 x 10^2^	<0.01
**25-hydroxyvitamin D**_**3**_ **(ng/ml)**	11.9 ± 4.2	12.1 ± 3.9	9.2 ± 3.5	0.6
**Cortisol (ng/ml)**	1.2 x 10^2^ ± 0.3 x 10^2^	1.4 x 10^2^ ± 0.6 x 10^2^	1.2 x 10^2^ ± 0.1 x 10^2^	0.38

SD, standard deviation

## Discussion

In our study, compared with healthy controls, men with psoriasis had significantly higher BDI scores, body mass indices, and serum concentrations of total cholesterol and interleukins 6 and 18; moreover, they had lower serum 25(OH)D_3_ concentrations. Depression severity correlated negatively with the 25(OH)D_3_ concentration and positively with psoriasis duration, the PASI, the percentage of body surface area affected by psoriatic lesions, and the interleukin-18 concentration.

In previous studies, we and other authors [5, 6, 11] showed a strong association between psoriasis and depressed mood. However, the association of depression with the severity and duration of psoriasis remains less clear [15–16]. The co-occurrence of psoriasis and depression may result from common mechanisms of these two conditions, and not solely from the psychological effect of skin lesions on patients’ mood. Our study examined some of the potential mechanisms that link psoriasis and depression.

In our study, compared to controls, patients with psoriasis had significantly higher BMIs and concentrations of total cholesterol; moreover, they had significantly higher concentrations of pro-inflammatory cytokines IL-6 and IL-18. These cytokines are implicated in the pathogenesis of psoriatic skin lesions. For example, overproduction of IL-6 in the skin stimulates T helper cells (Th)17 and inhibits the differentiation of regulatory T cells [25–26]. This, in turn, results in an increased production of pro-inflammatory cytokines, such as IL-17 and interferon (IFN) γ, which leads to keratinocyte proliferation and formation of psoriatic skin lesions. IL-18 is synthesized primarily by macrophages, but also by other immune cells and keratinocytes [27]. In the pathogenesis of psoriasis, IL-18 has two effects. First, IL-18 increases the production of IFN-γ, which promotes Th1-mediated responses. Second, IL-18 is involved in the recruitment and adhesion of immune cells to inflammatory sites [27]. Notably, patients with psoriasis have higher IL-18 concentrations in plasma than do healthy controls [28].

In line with previous studies, we found that patients with psoriasis had lower serum concentrations of 25(OH)D_3_ than did controls [29]. However, because people with high BMIs tend to have low 25(OH)D_3_ concentrations [30], the higher BMIs of patients with psoriasis in our study, compared with controls, can partly explain the difference in 25-(OH)D_3_ concentrations between these two groups.

We also studied the association of clinical and laboratory variables to depression in patients with psoriasis. Similarly to previous studies [9–10,12–14], we showed that psoriasis severity, measured with disease duration, PASI, and BSA, correlated with depression severity. Moreover, we studied whether both psoriasis and depression were associated with inflammation. Previous studies showed that patients with depression and no clinically important inflammatory conditions had high concentrations of IL-6 and IL-18 [17,31–32]. Higher concentrations of IL-6 and IL-18 were also independently associated with depressive disorders in patients after stroke [33], and depression severity correlated positively with serum concentrations of IL-6 and IL-18 in hemodialyzed patients [34]. Similarly, in our study, we found a statistically significant correlation between BDI scores and serum IL-18 concentrations in patients with psoriasis. Although the IL-6 concentration did not correlate with BDI scores in our patients, it tended to be higher (non-significantly) in patients with moderate depression than in patients who had only mild depression or no depression. The average patient in our study had mild depression (mean BDI score, 13±11), whereas most previous studies that showed increased serum IL-6 concentrations included patients with severe depression. We suppose that studies involving patients with psoriasis and severe depression could show that serum IL-6 concentrations and BDI scores are significantly correlated.

Our findings support the view that increased concentrations of pro-inflammatory cytokines may worsen depression. Some investigators suggested that depression might be caused by inflammation within the central nervous system (CNS) and by neurodegeneration [35]. Previous studies showed that inflammation, which can involve increased IL-6 concentrations, was associated with depression [36–37]. Moreover, treatments with agents against pro-inflammatory cytokines reduced depression in patients with chronic inflammatory conditions [38]. In patients with rheumatoid arthritis [39] and inflammatory bowel disease [40], anxiety and depression were associated with IL-17 concentrations. Activation of Th1 and Th17 cells, which leads to an increased production of IL-2, IFN γ, and IL-17, was also observed in depression [41–42]. Because IL-17 is implicated in the development of psoriatic lesions, it would be worthwhile examining the association between IL-17 and depression in psoriasis.

Evolutionarily, the association between inflammation and depression may be explained by the phenomenon of "sickness behavior". For example, a recuperating animal will withdraw from its environment to conserve energy needed for recovery [43]. On a biochemical level, pro-inflammatory cytokines disrupt kynurenine metabolism, and kynurenine concentrations in the blood were increased in people who attempted suicide [44]. Also, aberrant kynurenine metabolism generates neurotoxic quinolinic acid [43]. Moreover, plasma IL-18 concentrations have also been linked to the availability of μ-opioid receptor in patients with major depression [45].

In animal models, pro-inflammatory cytokines promoted the development of mental disorders by increasing the activity of the HPA axis [46]. Notably, hyperactivity of the HPA axis is one of the most consistent biological findings in patients with depression [47]. Based on these and similar observations [19, 20], we expected that depression severity would be related to cortisol concentrations in patients with psoriasis; however, this association was not significant in our study. The lack of an association between depression severity and serum cortisol concentrations might be because, in contrast to patients with depression, most patients with psoriasis have decreased serum concentrations of cortisol, which is due to disorders within the HPA axis and an impaired response to stress [20]. However, in our study, patients with psoriasis and controls had similar serum cortisol concentrations.

In our study, in patients with psoriasis, BDI scores correlated negatively with serum concentrations of 25(OH)D_3_. Vitamin D_3_ deficiency is implicated in the pathogenesis of both psoriasis and depression. Many studies showed that adequate systemic concentrations of vitamin-D_3_ metabolites enable normal differentiation and growth of keratinocytes [24, 29, 48]. In patients with psoriasis, low concentrations of 25(OH)D_3_ were associated with decreased counts of circulating T regulatory cells [48]. Thus, 25(OH)D_3_ may act as an immunomodulator and prevent excessive Th1 and Th17 responses, which are important in the pathogenesis of psoriasis. Notably, vitamin D_3_ supplementation improves skin lesions in some conditions [24]. In psoriasis, ultraviolet-B treatment alone or combined with vitamin D_3_ supplementation reduced psoriatic skin lesions and increased 25(OH)D_3_ concentration in serum [49–51].

Vitamin D_3_ deficiency may cause behavioral disorders in animals [52]. Similarly, absolute and relative deficiency of vitamin D_3_ may increase the risk of mood disorders in people [53]. Vitamin D_3_ deficiency is also associated with increased concentrations of inflammatory markers [23], which is consistent with the previously mentioned observations on the place of pro-inflammatory cytokines in the pathogenesis of depression [17, 54]. Vitamin D might be implicated in the development of depression, because vitamin D receptors are found in the CNS [55, 56], including the structures involved in mood control, such as the hippocampus and prefrontal cortex [57]. Moreover, in the brain, vitamin D is involved in the synthesis and release of serotonin, and thus vitamin D deficiency may disturb the function of serotoninergic brain systems, which could cause depression [58]. The negative correlation between serum 25(OH)D_3_ concentrations and depression severity in our patients with psoriasis supports previous observations, which indicate that vitamin D_3_ deficiency is implicated in the pathogenesis of both depression and psoriasis.

The skin is essential to the metabolism of vitamin D. First, the skin produces more than 95% of systemic vitamin D_3_. Moreover, the classical pathway of vitamin D_3_ activation to 1,25(OH)_2_D_3_ and 25(OH)D_3_, which involves the liver and kidneys or peripheral tissues, occurs also locally in the skin [59]. Importantly, the skin produces other biologically active metabolites of vitamin D_3_ via non-classical pathways (e.g. via CYP11A1) [59]. Thus, the non-classical pathways of vitamin D_3_ activation might be important in the pathogenesis of both psoriasis and depression [60].

Our study had some limitations. First, the study was cross-sectional, and thus we were unable to determine the exact sequence of events leading to inflammation, vitamin D_3_ deficiency, and development of psoriasis and depression. Second, the study included patients from a tertiary center only, and therefore the results cannot be generalized to the whole population of patients with psoriasis. Third, we analyzed few serum markers, and further studies should examine whether other factors, such as activation of oxidative and nitrosative stress pathways and other cytokines implicated in the pathogenesis of psoriasis (e.g., TNF-α, IL-17A, IL-12, IL-23), are associated with the development of depression in patients with psoriasis.

## Conclusions

Depression in patients with psoriasis is not only a psychological reaction to a chronic condition, but common mechanisms of depression and psoriasis, in particular inflammation and vitamin D_3_ deficiency, might be responsible for the co-occurrence of these two diseases. Our findings encourage further studies that should examine whether effective anti-inflammatory treatments or vitamin D_3_ supplementation can improve depression and psoriatic lesions in patients with psoriasis.
